# Value of muscle magnetic resonance imaging in the differential diagnosis of muscular dystrophies related to the dystrophin-glycoprotein complex

**DOI:** 10.1186/s13023-019-1242-y

**Published:** 2019-11-12

**Authors:** Zhiying Xie, Zhihao Xie, Meng Yu, Yiming Zheng, Chengyue Sun, Yilin Liu, Chen Ling, Ying Zhu, Wei Zhang, Jiangxi Xiao, Zhaoxia Wang, Yun Yuan

**Affiliations:** 1 Department of Neurology, Peking University First Hospital, 8 Xishiku Street, Xicheng District, Beijing, 100034 China; 2 Department of Epidemiology and Biostatistics, West China School of Public Health, Sichuan University, Chengdu, China; 3 Department of Radiology, Peking University First Hospital, Beijing, China

**Keywords:** Dystrophin-glycoprotein complex, Muscular dystrophy, Muscle magnetic resonance imaging, Differential diagnosis

## Abstract

**Background:**

Dystrophin-glycoprotein complex (DGC)-related muscular dystrophies may present similar clinical and pathological features as well as undetectable mutations thus being sometimes difficult to distinguish. We investigated the value of muscle magnetic resonance imaging (MRI) in the differential diagnosis of DGC-related muscular dystrophies and reported the largest series of Chinese patients with sarcoglycanopathies studied by muscle MRI.

**Results:**

Fifty-five patients with DGC-related muscular dystrophies, including 22 with confirmed sarcoglycanopathies, 11 with limb-girdle muscular dystrophy 2I (LGMD2I, *FKRP*-associated dystroglycanopathy), and 22 with dystrophinopathies underwent extensive clinical evaluation, muscle biopsies, genetic analysis, and muscle MRI examinations. Hierarchical clustering of patients according to the clinical characteristics showed that patients did not cluster according to the genotypes. No statistically significant differences were observed between sarcoglycanopathies and LGMD2I in terms of thigh muscle involvement. The concentric fatty infiltration pattern was observed not only in different sarcoglycanopathies (14/22) but also in LGMD2I (9/11). The trefoil with single fruit sign was observed in most patients with dystrophinopathies (21/22), and a few patients with sarcoglycanopathies (4/22) or LGMD2I (2/11). Hierarchical clustering showed that most patients with sarcoglycanopathies or LGMD2I can be distinguished from dystrophinopathies based on the concentric fatty infiltration pattern and trefoil with single fruit sign at the thigh level on muscle MRI.

**Conclusions:**

Muscle MRI at the thigh level potentially allows distinction of sarcoglycanopathies or *FKRP*-associated dystroglycanopathy from dystrophinopathies.

## Background

The dystrophin-glycoprotein complex (DGC) or the dystrophin-associated protein complex composed of the cytoplasmic dystrophin, syntrophin, α-dystrobrevin and neuronal nitric oxide synthase (nNOS), the transmembrane β-dystroglycan, α-, β-, γ- and δ-sarcoglycan (SG) and sarcospan, and the extracellular α-dystroglycan (α-DG), is essential for maintaining sarcolemma stability and muscle integrity [1]. Mutations in genes encoding DGC components can trigger sarcolemma instability and eventually lead to the development of muscular dystrophies [1]. DGC-related muscular dystrophies include dystrophinopathies caused by mutations in *DMD*, sarcoglycanopathies caused by mutations in *SGCG*, *SGCA*, *SGCB*, and *SGCD*, and dystroglycanopathies caused by mutations in *FKRP* and other genes associated with the O-mannose glycosylation pathway of α-DG [1–3].

Clinical phenotypes of DGC-related muscular dystrophies cover a wide and overlapping clinical spectrum [4–6]. Thus, differential diagnosis among different DGC-related muscular dystrophies cannot be made on clinical characteristics alone. Moreover, under certain conditions, concomitant reduction of dystrophin and sarcoglycans are observed in dystrophinopathies [7] and sarcoglycanopathies [8], and of dystrophin and glycosylated α-DG in dystroglycanopathies [9]; this hampers prediction of the primary genetic defect based on muscle immunoanalysis. Therefore, confirmatory diagnosis of DGC-related muscular dystrophies relies mainly on genetic testing. However, identifying the pathogenic variants responsible for DGC-related muscular dystrophies is complicated by non-coding sequence variants and structural variants, some of which remain undetectable [5]. Hence, it is necessary to establish other tests that can support differential diagnosis among different DGC-related muscular dystrophies.

Muscle magnetic resonance imaging (MRI) is increasingly used for diagnostic workup of neuromuscular disorders, because it contributes to recognition of muscle involvement patterns [10–12]. The distinctive patterns observed at the thigh level of DGC-related muscular dystrophies, including the trefoil with single fruit sign [13] and concentric fatty infiltration pattern [6], are highly specific for dystrophinopathies and *FKRP*-associated dystroglycanopathy, respectively. Thus, muscle MRI might potentially be useful for distinguishing these diseases, but this has not yet been tested. Moreover, there has been no comprehensive study on muscle MRI changes in Chinese patients with sarcoglycanopathies. Therefore, this study investigated the value of muscle MRI in differentiating among DGC-related muscular dystrophies and characterized the pattern of involvement on muscle MRI in Chinese patients with sarcoglycanopathies.

## Materials and methods

### Patients

Fifty-five patients who presented to Peking University First Hospital and were diagnosed with DGC-related muscular dystrophies based on clinical manifestations, muscle biopsy results, and relevant pathogenic variants were enrolled. Among these, 22 patients were confirmed to have dystrophinopathies, 22 to have sarcoglycanopathies, and 11 to have limb-girdle muscular dystrophy 2I (LGMD2I, *FKRP*-associated dystroglycanopathy). Immunohistochemistry was using monoclonal antibodies against DGC proteins, including dystrophin-N (amino-terminal), dystrophin-C (carboxyl-terminal), dystrophin-R (large central rod-domain), α-, β-, and γ-SG, and glycosylated α-DG [6, 14].

### Muscle MRI scans and interpretation

Except for conventional T1-weighted images (T1WI) of the lower leg muscles being obtained in 39 patients (patients 2, 5, 7–9, 11–14, 16, 17, 19–24, 26, 28, 32–35, 37–41, 43–45, 47–51, and 53–55), conventional T1WI of the pelvis and thigh muscles were obtained in all patients according to standard protocols [6]. All scans were independently interpreted by an experienced radiologist and a neurologist, who were blinded to the clinical information and molecular diagnosis during image review. The extent of fatty infiltration of individual muscles was graded on axial T1WI using a modified 0–5 Mercuri’s point scale [6, 15–18] as follows: stage 0, normal muscle appearance (score 0); stage 1, occasional scattered areas of increased density (score 1); stage 2a, numerous discrete areas of increased density less than 30% of the individual muscle volume (score 2); stage 2b, increased areas of confluent density, 30–60% of the individual muscle volume (score 3); stage 3, washed-out appearance due to increased areas of confluent density, more than 60% of the individual muscle volume (score 4);and stage 4, end-stage appearance, muscle entirely replaced by areas of confluent density (score 5).

### Statistical analysis

The median of patient age, age at onset, and disease duration, as well as frequency percentage for each score of fatty infiltration, were treated as descriptive statistics. The nonparametric Kruskal-Wallis test was used to compare the fatty infiltration of each individual muscle between patients with different DGC-related muscular dystrophies. If this test was statistically significant (*P* < 0.05), Nemenyi test was used for further pairwise multiple comparisons to locate the source of significance. Statistical analyses were performed using SPSS for Windows version 22.0.

For clustering of patients by clinical phenotypes, the following clinical characteristics were used as variables: age, age at onset, disease duration, creatine kinase (CK) value, walking ability, calf hypertrophy, tendon contractures, scapular winging, muscle pain, and muscle strength in muscle groups involving neck flexion, shoulder adduction, shoulder abduction, elbow extension, elbow flexion, grip muscle, hip flexion, hip adduction, hip abduction, knee extension, knee flexion, ankle dorsiflexion, and ankle plantar flexion. According to previous studies [5, 19], hierarchical clustering was analyzed and the scores of fatty infiltration in each individual muscle were represented as a heatmap using R software version 3.1.3. The Gower’s distance was used for clustering of patients. The R software automatically established the order of the patients in the heatmap and generated dendrograms linking patients with similar involvement; thus, if patients within one subtype of DGC-related muscular dystrophies showed relatively consistent muscle involvement overall, they could be clustered into one group.

## Results

### Patients

The clinical characteristics of patients with different DGC-related muscular dystrophies were listed in Additional file 1: Table S1 and their genetic and pathologic features in Additional file 2: Table S2 and Additional file 5: Figure S1. The detailed clinical, pathologic, and genetic features of 22 patients with sarcoglycanopathies (patients 1–22), and of 10 (patients 23–30) of 11 patients with LGMD2I (patients 23–33) have been reported in our previous works [6, 14]. Clinical phenotypes of patients with sarcoglycanopathies, LGMD2I or dystrophinopathies ranged from hyperCKemia or mildly affected to severe patients who had lost independent ambulation.

All 22 patients with sarcoglycanopathies showed variable reduction in expression of α-, β-, and γ-SG, and slightly to severely reduced dystrophin-N, -C, and -R in 14 of them (Additional file 5: Figure S1(c_2_–c_7_)). Patients with dystrophinopathies showed varying reduction or complete deficiency of dystrophin-N, -C, and -R, and variable reduction of α-, β-, and γ-SG ranging from a slight decrease to absence (Additional file 5: Figure S1(d_2_–d_7_)). Of 11 LGMD2I patients with two mutations in *FKRP*, 3 patients showed reduction of sarcoglycans and dystrophin (Additional file 5: Figure S1(b_2_–b_7_)). Nineteen patients with sarcoglycanopathies were found to have two mutations in *SGCA*, *SGCB*, or *SGCG*, but 3 patients were found to have only one mutation in *SGCA* or *SGCB*. The mutations identified in *SGCA*, *SGCB*, *SGCG*, *FKRP*, and *DMD* were of various types that were comprised of insertions/deletions (indels), single nucleotide variants (SNV), and deletions or duplications of one or more exons.

Hierarchical clustering of all 55 patients according to the clinical characteristics showed that patients did not cluster according to the genotypes (Additional file 6: Figure S2).

### Muscle MRI findings

**Fig. 1:**
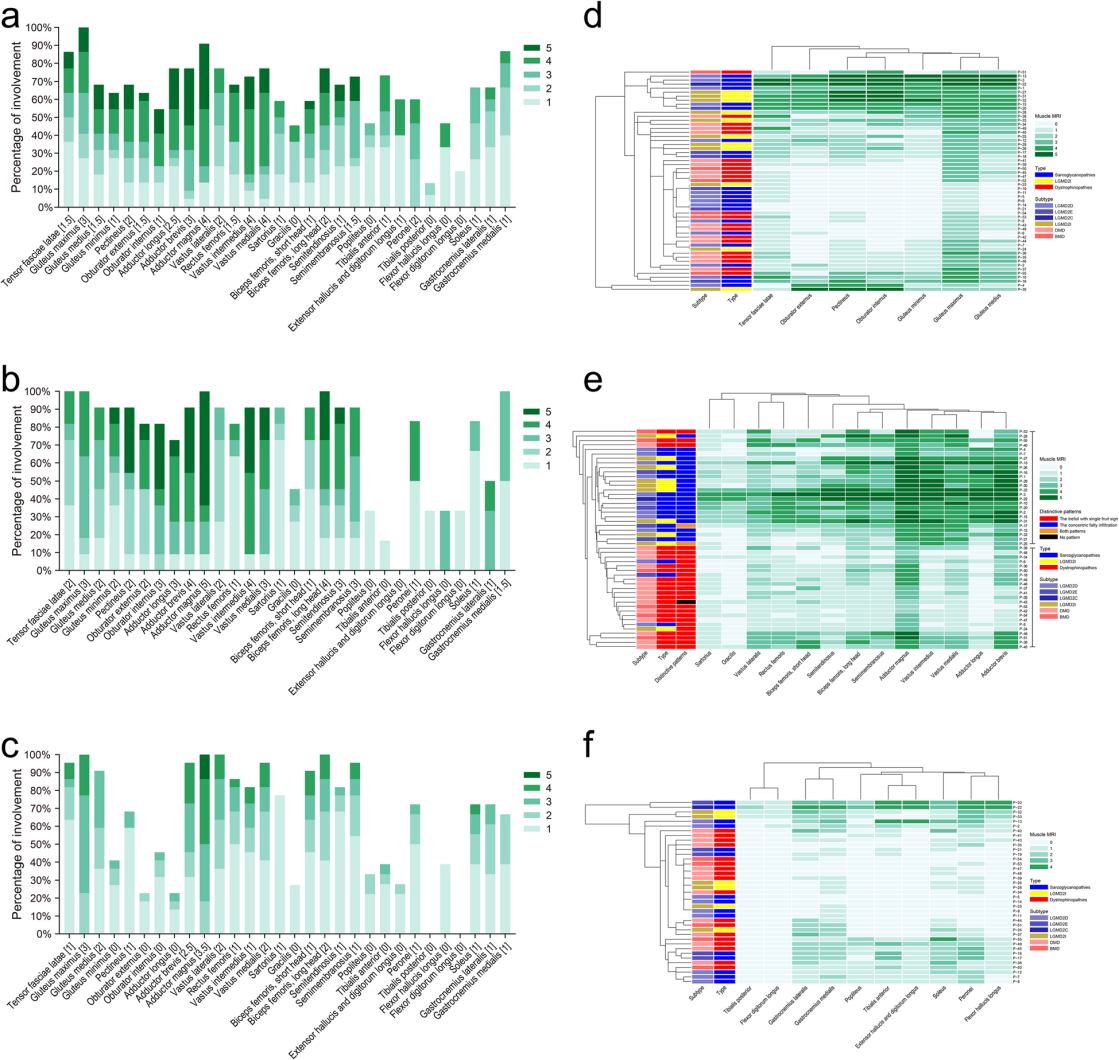
Summary of pelvis, thigh, and lower leg muscle involvement in DGC-related muscular dystrophies. **a**–**c** Frequency of fatty infiltration of the individual muscles was represented as a percentage of the all. Green bars represented the percentage of muscles affected for each score. The numbers within the square brackets indicated the median score for each muscle. **d** Hierarchical clustering of patients according to the individual pelvis muscles showing that patients did not cluster according to the genotypes. **e** In hierarchical clustering of patients according to the individual thigh muscles, 23 of the 26 clustered patients (upper group) showed the concentric fatty infiltration pattern, and 22 of the 23 clustered patients (lower group) showed the trefoil with single fruit sign. **f** Hierarchical clustering of patients according to the lower leg individual muscles showing that patients did not cluster according to the genotypes. DGC, dystrophin-glycoprotein complex; LGMD, limb-girdle muscular dystrophy; DMD, Duchenne muscular dystrophy; BMD, Becker muscular dystrophy

**Fig. 2:**
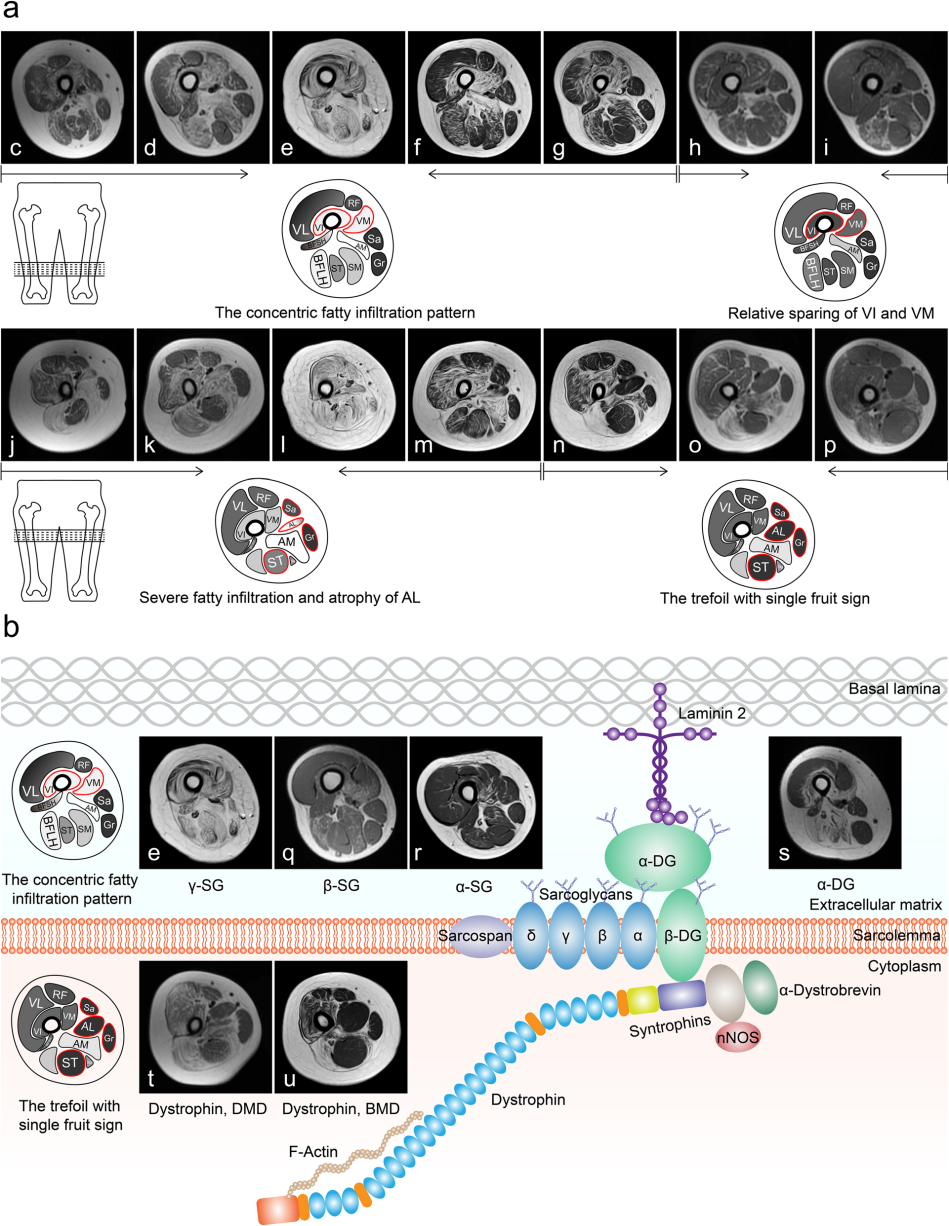
Distinctive patterns of fatty infiltration in DGC-related muscular dystrophies. **a** Representative cases of DGC-related muscular dystrophies showing distinctive patterns of muscle involvement. **c**–**g** and **q**–**s** Representative cases of sarcoglycanopathies and dystroglycanopathy showing the concentric fatty infiltration pattern. **o**, **p**, **t**, and **u** Representative cases of dystrophinopathies showing the trefoil with single fruit sign. **g** and **n** A representative case of sarcoglycanopathies showing both patterns. **j**–**m** Representative cases of sarcoglycanopathies and dystroglycanopathy showing severe fatty infiltration and relative sparing of the medial part of adductor longus. **c** and **j**, patient 1; **d** and **k**, patient 16; **e** and **l**, patient 22; **f** and **m**, patient 29; **g** and **n**, patient 17; **h** and **o**, patient 36; **i** and **p**, patient 50; **q**, patient 21; **r**, patient 4; **s**, patient 28; **t**, patient 38; **u**, patient 52. **b** Scheme of the DGC in skeletal muscle adapted from [1]. AL, adductor longus; Ab, adductor brevis; AM, adductor magnus; VL, vastus lateralis; RF, rectus femoris; VI, vastus intermedius; VM, vastus medialis; Sa, Sartorius; Gr, gracilis; BFSH, biceps femoris, short head; BFLH, biceps femoris, long head; ST, semitendinosus; SM, semimembranosus. DGC, dystrophin-glycoprotein complex; SG, sarcoglycan; DG, dystroglycan; LGMD, limb-girdle muscular dystrophy; DMD, Duchenne muscular dystrophy; BMD, Becker muscular dystrophy

The overall distribution and extent of fatty infiltration of the involved muscles were bilaterally symmetrical on axial T1WI (Fig. 2 and Additional file 7: Figure S3). The fatty infiltration percentage with each score and the median score for each muscle were shown in Fig. 1a–c. Percentages of different extent of fatty infiltration for each individual muscle in DGC-related muscular dystrophies were listed in Additional file 3: Table S3.

### Sarcoglycanopathies

At the pelvis level, the gluteus maximus muscle was the most affected, with 72.73% showing moderate or severe fatty infiltration, followed by the pectineus (54.55%), tensor fasciae latae (50.00%), gluteus medius (50.00%), obturator externus (50.00%), obturator internus (40.91%), and gluteus minimus (36.36%) muscles.

At the thigh level, the adductor magnus muscle was the most affected, with 68.18% showing severe fatty infiltration. The vastus intermedius (54.55%) and vastus medialis (54.55%) muscles had the next highest percentage, followed by the adductor brevis (45.45%), adductor longus (45.45%), and long head of biceps femoris (31.82%) muscles. The rectus femoris, semitendinosus, and short head of biceps femoris muscles showed mainly mild to moderate fatty infiltration. The vastus lateralis, semimembranosus, sartorius, and gracilis muscles were relatively spared and showed mainly mild fatty infiltration.

At the lower leg level, the peronei muscle was the most affected, with 60.00% showing moderate or severe fatty infiltration, followed by the gastrocnemius medialis (46.67%), tibialis anterior (40.00%), soleus (40.00%), and gastrocnemius lateralis (33.33%) muscles. The extensor halluces/digitorum longus, popliteus, flexor hallucis longus, tibialis posterior, and flexor digitorum longus muscles were completely or almost completely spared in the majority of patients.

### LGMD2I (*FKRP*-associated dystroglycanopathy)

At the pelvis level, the gluteus maximus muscle was the most affected, with 90.91% showing moderate or severe fatty infiltration, followed by the pectineus (81.82%), obturator internus (81.82%), gluteus medius (72.73%), obturator externus (72.73%), tensor fasciae latae (63.64%), and gluteus minimus (54.55%) muscles.

At the thigh level, the vastus intermedius muscle was the most involved, with 81.82% showing severe fatty infiltration. The other most severely affected muscles were the adductor magnus (72.73%), adductor brevis (63.64%), and long head of biceps femoris (54.55%) muscles. The adductor longus, semitendinosus, and vastus medialis muscles were equally involved and the percentage of severe fatty infiltration was 45.45%. The semimembranosus, short head of biceps femoris, and rectus femoris muscles showed mainly mild to moderate fatty infiltration. The vastus lateralis, sartorius, and gracilis muscles were relatively spared.

At the lower leg level, the gastrocnemius medialis and gastrocnemius lateralis muscles were the most affected, with 50.00% showing moderate or severe fatty infiltration, followed by the peronei (30.00%) and flexor hallucis longus (30.00%) muscles. The soleus, popliteus, tibialis anterior, extensor halluces/digitorum longus, tibialis posterior, and flexor digitorum longus muscles were completely or almost completely spared in all patients with LGMD2I.

### Dystrophinopathies

At the pelvis level, the tensor fasciae latae muscle was the most affected, with 100.00% showing moderate or severe fatty infiltration. The gluteus maximus (54.55%) and pectineus (31.82%) muscles had the next highest percentage. The gluteus medius, obturator internus, gluteus minimus, and obturator externus muscles were relatively spared.

At the thigh level, the adductor magnus muscle was the most affected, with 100.00% showing moderate to severe fatty infiltration. The adductor brevis (63.64%) and vastus lateralis (63.64%) muscles had the next highest percentage of moderate to severe fatty infiltration, followed by the long head of biceps femoris (59.09%), vastus medialis (54.55%), and short head of biceps femoris (45.45%) muscles. The vastus intermedius, semimembranosus, and rectus femoris muscles showed mainly mild to moderate fatty infiltration. The adductor longus, sartorius, gracilis and semitendinosus muscles were relatively spared.

At the lower leg level, the gastrocnemius lateralis muscle was the most affected, with 38.89% showing moderate or severe fatty infiltration, followed by the soleus (33.33%) and gastrocnemius medialis (27.78%) muscles. The peronei, popliteus, tibialis anterior, and extensor halluces/digitorum longus muscles were almost completely spared in all patients. The tibialis posterior, flexor hallucis longus, and flexor digitorum longus muscles were completely spared in all patients.

### Differences among different DGC-related muscular dystrophies

The Kruskal-Wallis test showed that fatty infiltration scores of the gluteus minimus (*P* = 0.008), pectineus (*P* = 0.005), obturator externus (*P* < 0.001), obturator internus (*P* = 0.008), adductor longus (*P* < 0.001), vastus intermedius (*P* = 0.005), semitendinosus (*P* = 0.017), tibialis anterior (*P* = 0.014), or extensor halluces/digitorum longus (*P* = 0.020) muscle differed significantly among sarcoglycanopathies, LGMD2I, and dystrophinopathies. Further pairwise multiple comparisons between sarcoglycanopathies, LGMD2I, and dystrophinopathies showed that: 1) fatty infiltration scores of the adductor longus muscle and of the obturator externus muscle in sarcoglycanopathies or LGMD2I were significantly higher than those in dystrophinopathies; 2) fatty infiltration scores of the gluteus minimus, pectineus, obturator internus, vastus intermedius, or semitendinosus muscle in LGMD2I was significantly higher than that in dystrophinopathies; 3) fatty infiltration scores of the tibialis anterior muscle and of the extensor halluces/digitorum longus muscle in sarcoglycanopathies were significantly higher than those in LGMD2I (Additional file 4: Table S4).

### Pattern recognition-based distinction of DGC-related muscular dystrophies

At the pelvis or lower leg level (Additional file 7: Figure S3), we did not observe any specific pattern of muscle involvement that was consistent within even one subtype of DGC-related muscular dystrophies. This was confirmed by hierarchical analysis that patients did not cluster according to the genotypes when using the scores given to the single pelvis or lower leg muscles as variables (Fig. 1d and f). However, there was a common pattern overall, that is, relative sparing of the lower leg muscles was obvious even in severe phenotypes, while the pelvis and thigh muscles were affected with more severe fatty infiltration in all subtypes of DGC-related muscular dystrophies.

Consistent features emerged from the evaluation of axial T1WI for pattern recognition at the thigh level (Table 1). Six patients (patients 5, 9, 11, 14, 19, and 23) with no or only slight involvement of the adductor magnus and/or long head of biceps femoris muscles showed no specific patterns; hence, they were not evaluated in pattern recognition or hierarchical clustering. The concentric fatty infiltration around the distal femoral diaphysis (Fig. 2c–g and q–s), consisting of severe fatty infiltration of the vastus intermedius and vastus medialis muscles, usually with relative sparing of the vastus lateralis, rectus femoris, and short head of biceps femoris muscles, was observed in most patients with different sarcoglycanopathies (14/17, 82.35%) and LGMD2I (9/10, 90.00%). The concentric fatty infiltration pattern was absent in patients with dystrophinopathies, partly as two-thirds of them showed relative sparing of the vastus intermedius and vastus medialis muscles (15/22, 68.18%).

**Table 1:** Pattern recognition in DGC-related muscular dystrophies at the thigh level

	In the proximal thigh			In the distal thigh	
	The trefoil with single fruit sign	Severe fatty infiltration and atrophy of adductor longus	Adductor longus medial sparing	The concentric fatty infiltration	Relative sparing of vastus intermedius and vastus medialis
LGMD2D	2/11 (18.18%)	8/11 (72.72%)	5/11 (45.45%)	9/11 (72.73%)	2/11 (18.18%)
LGMD2E	2/5 (40.00%)	2/5 (40.00%)	2/5 (40.00%)	4/5 (80.00%)	1/5 (20.00%)
LGMD2C	0/1 (0.00%)	1/1 (100.00%)	1/1 (100.00%)	1/1 (100.00%)	0/1 (0.00%)
Sarcoglycanopathies	4/17 (23.53%)	11/17 (64.71%)	8/17 (47.06%)	14/17 (82.35%)	3/17 (17.65%)
LGMD2I	2/10 (20.00%)	6/10 (60.00%)	5/10 (50.00%)	9/10 (90.00%)	1/10 (10.00%)
DMD	15/16 (93.75%)	0/16 (0.00%)	0/16 (0.00%)	0/16 (0.00%)	12/16 (75.00%)
BMD	6/6 (100.00%)	1/6 (16.67%)	0/6 (0.00%)	0/6 (0.00%)	3/6 (50.00%)
Dystrophinopathies	21/22 (95.45%)	1/22 (4.55%)	0/22 (0.00%)	0/22 (0.00%)	15/22 (68.18%)

Sarcoglycanopathies, LGMD2D, LGMD2E, and LGMD2C; *FKRP*-associated dystroglycanopathy, LGMD2I; Dystrophinopathies, DMD and BMD. DGC, dystrophin-glycoprotein complex; LGMD, limb-girdle muscular dystrophy; DMD, Duchenne muscular dystrophy; BMD, Becker muscular dystrophy

The trefoil with single fruit sign in the proximal thigh, consisting of three leaflets formed by relative sparing of the gracilis, sartorius, and adductor longus muscles and the single fruit formed by relative sparing of the semitendinosus muscle (Fig. 2o, p, t, and u), was observed in most patients with dystrophinopathies (21/22, 95.45%). In addition, 4 patients with sarcoglycanopathies and 2 patients with LGMD2I also showed this sign. Of these 6 patients (2 LGMD2D, 2 LGMD2E, and 2 LGMD2I), one LGMD2E (patient 17; Fig. 2g and n) and one LGMD2I (patient 25) showed both patterns. While 11 patients with sarcoglycanopathies (64.71%) and 6 patients with LGMD2I (60.00%) showed severe fatty infiltration and atrophy of the adductor longus muscle, only one patient with dystrophinopathy showed severe involvement of the adductor longus muscle; this difference was statistically significant. Less consistent but relative sparing of the adductor longus muscle was observed in the medial part compared to that in the lateral part in patients with sarcoglycanopathies (8/17, 47.06%) and LGMD2I (5/10, 50.00%) (Fig. 2j–m).

Twenty-six patients relatively clustered into one group and the remaining 23 patients relatively clustered into another group according to the results of hierarchical clustering (Fig. 1e), mainly because the dendrograms automatically linked patients with similar involvement and even in advanced disease, most patients (88.46%) in the upper group showed the consistent concentric fatty infiltration pattern, and most patients (95.65%) in the lower group showed the consistent trefoil with single fruit sign; therefore, they were relatively clustered into two different groups. According to the results of hierarchical clustering (Fig. 1e), 23 (88.46%) of the 26 clustered patients had sarcoglycanopathies or LGMD2I, and 19 (82.61%) of the 23 clustered patients had dystrophinopathies, suggesting that most patients with sarcoglycanopathies or *FKRP*-associated dystroglycanopathy can be distinguished from dystrophinopathies by muscle MRI at the thigh level according to the presence or absence of the concentric fatty infiltration pattern or trefoil with single fruit sign.

## Discussion

In the present study, we determined the value of muscle MRI in the differential diagnosis of DGC-related muscular dystrophies and reported the largest series of Chinese patients with sarcoglycanopathies studied by muscle MRI so far.

As in other studies [4–6], clinical phenotypes of patients with DGC-related muscular dystrophies in this study were markedly heterogeneous and had overlapping features, which was confirmed by hierarchical clustering that patients with DGC-related muscular dystrophies did not cluster according to the genotypes, making differential diagnosis among these diseases on clinical grounds alone impossible. Variable reduction of dystrophin and sarcoglycans was observed in most of our patients with sarcoglycanopathies or dystrophinopathies, and a slight reduction of sarcoglycans and dystrophin was observed in a few patients with LGMD2I, suggesting that it is sometimes incorrect to predict the primary genetic defect based on muscle immunoanalysis due to secondary reduction of other DGC proteins [7–9]. The various types of mutations in *SGCA*, *SGCB*, *SGCG*, *FKRP*, and *DMD* contributed in part to the marked heterogeneity of clinical phenotypes, because different types of mutations are associated with different expression of DGC-associated proteins [6, 14] and may be associated with different degrees of protein dysfunction.

Our findings in sarcoglycanopathies that the gluteus maximus muscle was the most affected and the gluteus minimus muscle was the least affected at the pelvis level were contrary to the findings of previous studies [5, 20]. However, degree of fatty infiltration of the pectineus, gluteus medius, and obturator externus muscles was similar to the study by Tasca et al. [5]. At the lower leg level, relative sparing of the tibialis posterior and flexor digitorum longus muscles was consistent with the study by Tasca et al. [5], although the extensor halluces/digitorum longus and tibialis anterior muscles were not as severe as in the study by Tasca et al. [5]. As in the studies by Willis et al. [17, 18], the gastrocnemius medialis and gastrocnemius lateralis muscles were also affected with more severe fatty infiltration compared with the other lower leg muscles in LGMD2I, but relative sparing of the soleus muscle in our study was inconsistent with the mild involvement observed in the studies by Willis et al. [17, 18]. Similar to the study by Polavarapu et al. [21], the gastrocnemius lateralis and gastrocnemius medialis muscles were also the most affected in dystrophinopathies, and the little difference between dystrophinopathies and LGMD2I in our study was the mild involvement of the soleus muscle at the lower leg level.

Almost consistent with the previous studies [5, 17, 18, 20, 22], relative sparing of the lower leg muscles and more severe fatty infiltration of the pelvis and thigh muscles were observed in all subtypes of DGC-related muscular dystrophies. Hierarchical clustering of patients according to the pelvis or lower leg individual muscles revealed that patients did not cluster according to the genotypes, indicating that muscle MRI at the pelvis or lower leg level may not have any differential value in distinguishing different DGC-related muscular dystrophies, mainly because of no consistent pattern of muscle involvement at the pelvis or lower leg level.

At the thigh level, the muscle most severely affected by fatty infiltration in sarcoglycanopathies was the adductor magnus muscle, followed by the vastus intermedius and vastus medialis muscles. These findings were almost consistent with the previously reported selective muscle involvement wherein the adductor magnus and vastus intermedius muscles, with or without the vastus medialis muscle, were severely affected in LGMD2C [23], LGMD2D [20, 22, 24], LGMD2E [5], and LGMD2F [5]. Similar to previous studies [5, 20, 23, 24], the vastus lateralis, sartorius, and gracilis muscles were relatively spared in sarcoglycanopathies in our study. A pattern of selective muscle involvement, i.e., the concentric fatty infiltration pattern, appears to be distinctive for sarcoglycanopathies, because it was observed in most patients with various sarcoglycanopathies, including the most severely affected patients, consistent with the previously reported pattern observed in LGMD2C–2F proposed by Tasca et al. [5] and was also noted in other reports of LGMD2C [23] and LGMD2D [20, 24]. Our findings varied slightly from the pattern proposed by Tasca et al. [5], relative sparing of the medial part of the adductor longus muscle in sarcoglycanopathies was less frequent in our study than in the study of Tasca et al. [5], although it was a rather peculiar pattern.

As no statistically significant differences in the individual muscles could be found between sarcoglycanopathies and LGMD2I at the thigh level, the concentric fatty infiltration pattern was also apparent in most patients with LGMD2I. Additionally, relative sparing of the medial part of the adductor longus muscle was observed in nearly half of patients with sarcoglycanopathies or LGMD2I. These two distinctive patterns, particularly the concentric fatty infiltration pattern, seems to be homogeneous among LGMD2I [6] and different sarcoglycanopathies, which supports the idea that as sarcoglycans and glycosylated α-DG are relatively close to each other on the sarcolemma (Fig. 2b), any defects in these proteins likely cause damage to similar target muscles [5]. As we previously described [6], the concentric fatty infiltration pattern was rarely observed in other muscular dystrophies including dystrophinopathies [13], laminopathies [25], congenital muscular dystrophy with rigid spine syndrome [26], collagen VI-related myopathy [11], *RYR1*-related myopathies [27], Emery-Dreifuss muscular dystrophy [28], and dysferlinopathy [12]. Therefore, this pattern could be useful in the differential diagnosis between sarcoglycanopathies or LGMD2I and other muscular dystrophies.

The major difference we found between sarcoglycanopathies or LGMD2I and dystrophinopathies at the thigh level was that fatty infiltration of the adductor longus muscle in sarcoglycanopathies or LGMD2I was more severe than that in dystrophinopathies, which was a major contribution to the trefoil with single fruit sign [13] observed in most patients with dystrophinopathies. Although only a few patients with sarcoglycanopathies or LGMD2I showed the trefoil with single fruit sign, these overlapping patterns may indicate a common pathophysiology for different DGC-related muscular dystrophies: different defects in DGC proteins eventually lead to instability of the sarcolemma [1], which might result in similarities among the affected muscles.

Hierarchical clustering of patients according to the individual thigh muscles revealed that most patients with sarcoglycanopathies or LGMD2I can be distinguished from dystrophinopathies by muscle MRI according to the presence or absence of the concentric fatty infiltration pattern or trefoil with single fruit sign, indicating that these two distinctive patterns are of high differential value in distinguishing sarcoglycanopathies or *FKRP*-associated dystroglycanopathy from dystrophinopathies.

In conclusion, a distinctive pattern of muscle involvement, the concentric fatty infiltration pattern around the distal femoral diaphysis, is shared by the different sarcoglycanopathies and *FKRP*-associated dystroglycanopathy. While differential diagnosis of DGC-related muscular dystrophies based on clinical phenotypes or muscle immunoanalysis is problematic, most patients with sarcoglycanopathies or *FKRP*-associated dystroglycanopathy can be distinguished from dystrophinopathies by muscle MRI at the thigh level based on the concentric fatty infiltration pattern and trefoil with single fruit sign.

## Supplementary information

**Additional file 1: Table S1.** Clinical features of patients with DGC-related muscular dystrophies.

**Additional file 2: Table S2.** Genetic and pathologic features of patients with DGC-related muscular dystrophies.

**Additional file 3: Table S3.** Percentages of different extent of fatty infiltration for each individual muscle in DGC-related muscular dystrophies.

**Additional file 4: Table S4.** Further pairwise multiple comparisons between patients with different DGC-related muscular dystrophies.

**Additional file 5: Figure S1.** Pathologic features of patients with DGC-related muscular dystrophies. (b_1_, c_1_, and d_1_) Hematoxylin-eosin staining showing a dystrophic pattern in patients 32, 12, and 48. (a_2_–a_8_) A normal control showing positive staining of sarcoglycans, dystrophin, and glycosylated α-DG. (b_2_–b_8_) Patient 32 with LGMD2I showing complete deficiency of glycosylated α-DG, reduction of β-SG and dystrophin-C and -R, and positive staining of α-SG, γ-SG, and dystrophin-N. (c_2_–c_7_) Patient 12 with LGMD2D showing complete deficiency of α-SG and β-SG, reduction of γ-SG, and slight reduction of dystrophin. (d_2_–d_7_) Patient 48 with DMD showing complete deficiency of dystrophin-N and -C, severe reduction of dystrophin-R and β-SG, and slight reduction of α- and γ-SG. Hematoxylin-eosin staining (200× magnification); sarcoglycans, dystrophin, and glycosylated α-DG (400× magnification). DGC, dystrophin-glycoprotein complex; DG, dystroglycan; SG, sarcoglycan; LGMD, limb-girdle muscular dystrophy; DMD, Duchenne muscular dystrophy.

**Additional file 6: Figure S2.** Hierarchical clustering of patients according to the clinical characteristics showing that patients did not cluster according to the genotypes. DGC, dystrophin-glycoprotein complex; LGMD, limb-girdle muscular dystrophy; DMD, Duchenne muscular dystrophy; BMD, Becker muscular dystrophy.

**Additional file 7: Figure S3.** Examples of muscle fatty infiltration at the pelvis and lower leg level in DGC-related muscular dystrophies. a, patient 10, LGMD2D; b, patient 16, LGMD2E; c, patient 24, LGMD2I; d, patient 47, DMD; e, patient 54, BMD; f, patient 13, LGMD2D; g, patient 20, LGMD2E; h, patient 33, LGMD2I; i, patient 45, DMD; j, patient 55, BMD. DGC, dystrophin-glycoprotein complex; LGMD, limb-girdle muscular dystrophy; DMD, Duchenne muscular dystrophy; BMD, Becker muscular dystrophy.

## Data Availability

The datasets used and/or analyzed during this study are available from the corresponding author upon request.

## Abbreviations

BMD: Becker muscular dystrophy
DGC: Dystrophin-glycoprotein complex
DMD: Duchenne muscular dystrophy
LGMD: Limb-girdle muscular dystrophy
MRI: Magnetic resonance imaging
nNOS: Neuronal nitric oxide synthase
SG: Sarcoglycan
SNV: Single nucleotide variants
T1WI: T1-weighted images
α-DG: dystroglycan
