# Supplementary material for: Value of muscle magnetic resonance imaging in the differential diagnosis of muscular dystrophies related to the dystrophin-glycoprotein complex
Source: Orphanet J Rare Dis. 2019 Nov 12;14:250. doi: 10.1186/s13023-019-1242-y (PMC6865054; doi:10.1186/s13023-019-1242-y)
Supplement: Supplementary file 1 — Additional file 1: Table S1. Clinical features of patients with DGC-related muscular dystrophies. [file 13023_2019_1242_MOESM1_ESM.docx]

**Table S1**. Clinical features of patients with DGC-related muscular dystrophies.

| Patients | Diagnosis | Diagnosis subtype | Age, years/sex | Age at onset, years | Disease duration, years | Symptom(s) at onset | CK (IU/L) | Walking ability* | Distribution of weakness | Motor signs and symptoms | | | |
| --- | --- | --- | --- | --- | --- | --- | --- | --- | --- | --- | --- | --- | --- |
|  |  |  |  |  |  |  |  |  |  | Calf hypertrophy | Tendon contractures | Scapular winging | Muscle pain |
| P-1 | Sarcoglycanopathy | LGMD2D + CMT1A | 9.9/M | 2 | 7.9 | delayed motor milestones | 18559 | 3 | Proximal | + | + | – | – |
| P-2 | Sarcoglycanopathy | LGMD2D | 9.2/F | 5 | 4.2 | difficulties in running | 10300 | 2 | Proximal | + | – | – | – |
| P-3 | Sarcoglycanopathy | LGMD2D | 27.4/F | 11 | 16.4 | proximal lower limb weakness | 763 | 5 | Generalized | – | – | – | – |
| P-4 | Sarcoglycanopathy | LGMD2D | 10.3/F | 2 | 8.3 | exercise intolerance | 17690 | 3 | Proximal | + | + | – | – |
| P-5 | Sarcoglycanopathy | LGMD2D | 13.5/M | 10.5 | 3 | post-exercise muscle pain | 1770.7 | 1 | None | – | – | – | + |
| P-6 | Sarcoglycanopathy | LGMD2D | 7.4/M | 2 | 5.4 | difficulties in running and jumping | 8000 | 2 | Proximal | – | – | – | – |
| P-7 | Sarcoglycanopathy | LGMD2D | 11.8/F | 9.8 | 2 | difficulties in running and climbing stairs | 5355 | 2 | Proximal | – | – | + | – |
| P-8 | Sarcoglycanopathy | LGMD2D | 7.2/F | 2.2 | 5 | difficulties in jumping and climbing stairs | 12400 | 2 | Proximal | – | – | – | – |
| P-9 | Sarcoglycanopathy | LGMD2D | 7.7/M | 3.7 | 4 | post-exercise muscle pain | 8560 | 1 | None | + | – | – | + |
| P-10 | Sarcoglycanopathy | LGMD2D | 9.6/M | 7 | 2.6 | proximal lower limb weakness | 13814 | 3 | Proximal | + | + | – | – |
| P-11 | Sarcoglycanopathy | LGMD2D | 8.8/M | 2 | 6.8 | post-exercise muscle pain | 2650 | 1 | None | + | – | – | + |
| P-12 | Sarcoglycanopathy | LGMD2D | 9/M | 1 | 8 | difficulties in climbing stairs | 10347 | 2 | Proximal | – | – | – | + |
| P-13 | Sarcoglycanopathy | LGMD2D | 23.5/F | 11 | 12.5 | difficulties in running and climbing stairs | 2184 | 4 | Generalized | – | – | + | – |
| P-14 | Sarcoglycanopathy | LGMD2D | 25.5/F | 10 | 15.5 | post-exercise muscle pain | 400 | 1 | None | – | – | – | + |
| P-15 | Sarcoglycanopathy | LGMD2D | 12.2/F | 8.2 | 4 | abnormal gait | 3950 | 3 | Generalized | + | + | + | – |
| P-16 | Sarcoglycanopathy | LGMD2E | 12/M | 3 | 9 | early fatigue | 8022 | 3 | Proximal | + | + | – | – |
| P-17 | Sarcoglycanopathy | LGMD2E | 14.4/M | 9.5 | 4.9 | myalgias and exercise intolerance | 10000 | 2 | Proximal | + | + | – | + |
| P-18 | Sarcoglycanopathy | LGMD2E | 9.1/F | 8 | 1.1 | proximal lower limb weakness | 7873 | 2 | Proximal | – | – | – | – |
| P-19 | Sarcoglycanopathy | LGMD2E | 3.2/M | 0.8 | 2.4 | asymptomatic hyperCKemia | 35120 | 2 | None | + | – | – | – |
| P-20 | Sarcoglycanopathy | LGMD2E | 12.9/M | 2 | 10.9 | delayed motor milestones | 2247 | 4 | Generalized | – | – | – | – |
| P-21 | Sarcoglycanopathy | LGMD2E | 10.7/M | 5 | 5.7 | abnormal gait | 10085 | 2 | Proximal | + | + | – | – |
| P-22 | Sarcoglycanopathy | LGMD2C | 29/F | 7.5 | 21.5 | frequent falls | 842 | 5 | Generalized | – | + | + | – |
| P-23 | Dystroglycanopathy | LGMD2I | 3/M | 1.5 | 1.5 | asymptomatic hyperCKemia | 12739 | 1 | None | + | – | – | – |
| P-24 | Dystroglycanopathy | LGMD2I | 4/M | 2 | 2 | hyperCKemia; difficulties in running and jumping | 23131 | 1 | Proximal | + | – | – | – |
| P-25 | Dystroglycanopathy | LGMD2I | 14/M | 8 | 6 | difficulties in running | 5967 | 2 | Proximal | – | – | – | – |
| P-26 | Dystroglycanopathy | LGMD2I | 13/M | 3 | 10 | proximal lower limb weakness | 7800 | 2 | Proximal | + | – | – | – |
| P-27 | Dystroglycanopathy | LGMD2I | 34/M | 14 | 20 | abnormal gait | 4000 | 2 | Proximal | + | + | + | – |
| P-28 | Dystroglycanopathy | LGMD2I | 36/F | 29 | 7 | difficulties in climbing stairs | 4180 | 2 | Proximal | – | – | – | – |
| P-29 | Dystroglycanopathy | LGMD2I | 16/F | 5 | 5 | proximal lower limb weakness | 2220 | 3 | Proximal | + | – | – | – |
| P-30 | Dystroglycanopathy | LGMD2I | 24/F | 12 | 12 | difficulties in running; exercise intolerance | 964 | 3 | Proximal | – | + | – | – |
| P-31 | Dystroglycanopathy | LGMD2I | 16/F | 2 | 14 | difficulties in running; frequent falls | 5433 | 4 | Generalized | + | + | – | – |
| P-32 | Dystroglycanopathy | LGMD2I | 37/F | 17 | 20 | difficulties in climbing stairs | 643 | 4 | Generalized | + | + | – | – |
| P-33 | Dystroglycanopathy | LGMD2I | 9.2/F | 1.5 | 7.7 | hyperCKemia; Gowers' sign | 6673 | 2 | Generalized | – | + | – | – |
| P-34 | Dystrophinopathy | DMD | 8.1/M | 4 | 4.1 | difficulties in running and jumping | 12000 | 1 | Proximal | + | + | – | – |
| P-35 | Dystrophinopathy | DMD | 9.1/M | 3.5 | 5.6 | Frequent falls; Gowers' sign | 7444 | 2 | Generalized | + | + | – | – |
| P-36 | Dystrophinopathy | DMD | 7.5/M | 3 | 4.5 | Frequent falls; difficulties in running and jumping | 6717 | 2 | Generalized | + | + | – | + |
| P-37 | Dystrophinopathy | DMD | 6.9/M | 3 | 3.9 | delayed motor milestones | 10808 | 3 | Proximal | + | + | – | + |
| P-38 | Dystrophinopathy | DMD | 9/M | 3 | 6 | Frequent falls; Gowers' sign | 13919 | 3 | Proximal | + | + | – | – |
| P-39 | Dystrophinopathy | DMD | 8.4/M | 5 | 3.4 | Gowers' sign | 15150 | 3 | Generalized | + | + | – | – |
| P-40 | Dystrophinopathy | DMD | 7.1/M | 3.5 | 3.6 | delayed motor milestones | 7856 | 2 | Proximal | + | + | – | – |
| P-41 | Dystrophinopathy | DMD | 7.6/M | 3.6 | 4 | difficulties in running and jumping | 8918 | 2 | Proximal | + | + | – | – |
| P-42 | Dystrophinopathy | DMD | 3.5/M | 2 | 1.5 | delayed motor milestones | 7156 | 1 | None | – | – | – | – |
| P-43 | Dystrophinopathy | DMD | 5.2/M | 2 | 3.2 | delayed motor milestones | 27157 | 2 | Proximal | + | – | – | + |
| P-44 | Dystrophinopathy | DMD | 10.2/M | 4.5 | 5.7 | myalgias; proximal lower limb weakness | 12051 | 2 | Proximal | – | + | – | + |
| P-45 | Dystrophinopathy | DMD | 8.6/M | 4 | 4.6 | difficulties in running and jumping | 6157 | 3 | Proximal | + | + | – | – |
| P-46 | Dystrophinopathy | DMD | 7.8/M | 2 | 5.8 | delayed motor milestones | 12670 | 3 | Generalized | + | + | – | – |
| P-47 | Dystrophinopathy | DMD | 6.8/M | 2.5 | 4.3 | delayed motor milestones; post-exercise muscle pain | 8000 | 1 | None | + | + | – | + |
| P-48 | Dystrophinopathy | DMD | 4.5/M | 2 | 2.5 | delayed motor milestones | 20100 | 2 | Proximal | + | – | – | – |
| P-49 | Dystrophinopathy | DMD | 9.9/M | 5 | 4.9 | difficulties in running, jumping and climbing stairs | 6785 | 3 | Generalized | + | + | – | – |
| P-50 | Dystrophinopathy | BMD | 11.8/M | 7.5 | 4.3 | difficulties in running and climbing stairs | 3031 | 2 | Proximal | + | + | – | – |
| P-51 | Dystrophinopathy | BMD | 12.5/M | 9 | 3.5 | proximal lower limb weakness | 3826 | 2 | Proximal | + | + | – | – |
| P-52 | Dystrophinopathy | BMD | 19/M | 18 | 1 | Frequent falls | 4783 | 2 | Proximal | + | + | – | – |
| P-53 | Dystrophinopathy | BMD | 10.6/M | 7.7 | 2.9 | proximal lower limb weakness | 2830 | 2 | Proximal | + | + | – | – |
| P-54 | Dystrophinopathy | BMD | 6.8/M | 3.7 | 3.1 | hyperCKemia; post-exercise muscle pain | 8474 | 1 | None | + | – | – | + |
| P-55 | Dystrophinopathy | BMD | 16.8/M | 8.5 | 8.3 | post-exercise muscle pain; proximal lower limb weakness | 4479 | 3 | Generalized | + | + | – | – |

*, Walking ability was scored as follows: 1, asymptomatic hyperCKemia with or without exercise-induced myalgia; 2, running with difficulties; 3, unable to run; 4, ambulant with support; 5, non-ambulant. DGC, dystrophin-glycoprotein complex; LGMD, limb-girdle muscular dystrophy; CMT1A, Charcot-Marie-Tooth 1A; DMD, Duchenne muscular dystrophy; BMD, Becker muscular dystrophy; F, female; M, male; CK, creatine kinase.
