# Supplementary material for: Value of muscle magnetic resonance imaging in the differential diagnosis of muscular dystrophies related to the dystrophin-glycoprotein complex
Source: Orphanet J Rare Dis. 2019 Nov 12;14:250. doi: 10.1186/s13023-019-1242-y (PMC6865054; doi:10.1186/s13023-019-1242-y)
Supplement: Supplementary file 2 — Additional file 2: Table S2. Genetic and pathologic features of patients with DGC-related muscular dystrophies. [file 13023_2019_1242_MOESM2_ESM.docx]

**Table S2**. Genetic and pathologic features of patients with DGC-related muscular dystrophies.

| Patients | Gene | Molecular genetics (mutations) | | Pathological pattern | Immunohistochemistry staining | | | | | | |
| --- | --- | --- | --- | --- | --- | --- | --- | --- | --- | --- | --- |
|  |  | Allele 1 | Allele 2 |  | α-SG | β-SG | γ-SG | dystrophin-N | dystrophin-C | dystrophin-R | glycosylated α-DG |
| P-1 | *SGCA* | c.101G>A, p.R34H | c.218C>G, p.P73R | Dystrophic | – | – | – | +/– | +/– | +/– | NA |
|  | *PMP22* | Exons 1-5 duplication | - |  |  |  |  |  |  |  |  |
| P-2 | *SGCA* | c.313-2A>G, splicing | c.95T>C, p.V32A | Dystrophic | – | – | + | +++ | +++ | +++ | NA |
| P-3 | *SGCA* | c.424A>G, p.S142G | unknown | Dystrophic | +/– | +/– | +/– | +++ | +++ | +++ | NA |
| P-4 | *SGCA* | c.889delC, p.L298Cfs*23 | c.292C>T, p.R98C | Dystrophic | – | – | + | +++ | +++ | +++ | NA |
| P-5 | *SGCA* | c.661C>T, p.R221C | c.320C>T, p.A107V | Mild | ++ | +++ | +++ | +++ | +++ | +++ | NA |
| P-6 | *SGCA* | c.233_234delinsGA, p.Y78* | c.371T>C, p.I124T | Dystrophic | – | +/– | + | ++ | ++ | +++ | NA |
| P-7 | *SGCA* | Exons 1-7 duplication | c.229C>T, p.R77C | Dystrophic | +/– | +/– | + | ++ | ++ | ++ | NA |
| P-8 | *SGCA* | Exons 4-8 deletion | c.234C>A, p.Y78* | Dystrophic | – | – | + | +++ | +++ | +++ | NA |
| P-9 | *SGCA* | c.956+2T>C, splicing | c.662G>A, p.R221H | Mild | ++ | + | ++ | +++ | +++ | +++ | NA |
| P-10 | *SGCA* | c.427C>T, p.H143Y | c.229C>T, p.R77C | Dystrophic | +/– | +/– | ++ | + | + | +++ | NA |
| P-11 | *SGCA* | c.662G>A, p.R221H | c.956G>A, p.R319K | Mild | ++ | ++ | +++ | ++ | ++ | ++ | NA |
| P-12 | *SGCA* | Exons 7-8 deletion | c.218C>T, p.P73L | Dystrophic | – | – | + | ++ | ++ | ++ | NA |
| P-13 | *SGCA* | c.1A>G, p.0? | c.1A>G, p.0? (hom) | Dystrophic | +/– | – | + | ++ | +++ | +++ | NA |
| P-14 | *SGCA* | c.662G>A, p.R221H | c.95T>C, p.V32A | Mild | +++ | ++ | +++ | +++ | +++ | +++ | NA |
| P-15 | *SGCA* | c.409G>A, p.E137K | c.687delT, p.L230Cfs*18 | Dystrophic | +/– | – | – | ++ | ++ | +++ | NA |
| P-16 | *SGCB* | c.551A>G, p.Y184C | Exons 5-6 deletion | Dystrophic | ++ | + | ++ | +++ | ++ | ++ | NA |
| P-17 | *SGCB* | c.334C>T, p.Q112* | unknown | Dystrophic | + | – | + | ++ | ++ | +/– | NA |
| P-18 | *SGCB* | c.29_33delAACAG, p.E10Afs*13 | c.29_33delAACAG, p.E10Afs*13 (hom) | Dystrophic | +/– | – | +/– | ++ | ++ | ++ | NA |
| P-19 | *SGCB* | c.273_292del, p.I92* | c.273_292del, p.I92* (hom) | Dystrophic | – | – | – | + | +++ | +++ | NA |
| P-20 | *SGCB* | c.29_33delAACAG, p.E10Afs*13 | c.366_367delTT, p.Y123* | Dystrophic | – | – | +/– | ++ | +++ | +++ | NA |
| P-21 | *SGCB* | c.543C>A, p.S181R | unknown | Dystrophic | ++ | – | +++ | ++ | +++ | +++ | NA |
| P-22 | *SGCG* | c.320C>T, p.S107L | c.320C>T, p.S107L (hom) | Dystrophic | + | +/– | – | +++ | +++ | +++ | NA |
| P-23 | *FKRP* | c.204_206delCTC, p.S69del | c.545A>G, p.Y182C | Dystrophic | ++ | +++ | +++ | ++ | +++ | ++ | NA |
| P-24 | *FKRP* | c.1263C>A, p.Y421* | c.545A>G, p.Y182C | Dystrophic | +++ | +++ | +++ | +++ | +++ | +++ | – |
| P-25 | *FKRP* | c.545A>G, p.Y182C | c.545A>G, p.Y182C (hom) | Dystrophic | +++ | +++ | +++ | +++ | +++ | +++ | NA |
| P-26 | *FKRP* | c.545A>G, p.Y182C | c.1067T>C, p.I356T | Dystrophic | +++ | +++ | +++ | +++ | +++ | +++ | – |
| P-27 | *FKRP* | c.545A>G, p.Y182C | c.545A>G, p.Y182C (hom) | Mild | +++ | +++ | +++ | +++ | +++ | +++ | +/– |
| P-28 | *FKRP* | c.545A>G, p.Y182C | c.545A>G, p.Y182C (hom) | Mild | +++ | +++ | +++ | +++ | +++ | +++ | – |
| P-29 | *FKRP* | c.545A>G, p.Y182C | c.1027G>T, p.E343* | Dystrophic | +++ | +++ | +++ | +++ | +++ | +++ | +/– |
| P-30 | *FKRP* | c.545A>G, p.Y182C | c.545A>G, p.Y182C (hom) | Dystrophic | +++ | +++ | +++ | +++ | +++ | +++ | – |
| P-31 | *FKRP* | c.545A>G, p.Y182C | c.534G>T, p.W178C | Dystrophic | +++ | +++ | +++ | +++ | +++ | +++ | NA |
| P-32 | *FKRP* | c.545A>G, p.Y182C | c.160C>T, p.R54W | Dystrophic | +++ | + | +++ | +++ | ++ | ++ | – |
| P-33 | *FKRP* | c.250G>T, p.D84Y | c.776dupG, p.E260Rfs*130 | Dystrophic | +++ | ++ | +++ | ++ | ++ | ++ | + |
| P-34 | *DMD* | Exons 3-11 deletion | - | Dystrophic | +++ | + | +++ | – | + | ++ | NA |
| P-35 | *DMD* | Exons 53-60 deletion | - | Dystrophic | + | +/– | +++ | – | – | – | NA |
| P-36 | *DMD* | Exons 8-12 deletion | - | Dystrophic | +++ | ++ | +++ | – | +/– | – | NA |
| P-37 | *DMD* | Exons 45-54 deletion | - | Dystrophic | +++ | + | +++ | + | +/– | ++ | NA |
| P-38 | *DMD* | Exons 18-41 deletion | - | Dystrophic | ++ | + | ++ | +/– | +/– | – | NA |
| P-39 | *DMD* | Exons 47-50 deletion | - | Dystrophic | +++ | +/– | +++ | – | +/– | +/– | NA |
| P-40 | *DMD* | Exon 51 deletion | - | Dystrophic | ++ | + | ++ | – | – | + | NA |
| P-41 | *DMD* | Exon 64 deletion | - | Dystrophic | ++ | +/– | ++ | – | – | +/– | NA |
| P-42 | *DMD* | Exons 46-52 deletion | - | Dystrophic | ++ | +/– | +++ | – | – | ++ | NA |
| P-43 | *DMD* | Exons 48-52 deletion | - | Dystrophic | + | – | + | – | – | – | NA |
| P-44 | *DMD* | Exon 45 deletion | - | Dystrophic | +++ | + | +++ | +/– | +/– | + | NA |
| P-45 | *DMD* | Exon 52 duplication | - | Dystrophic | +++ | +/– | +++ | – | – | +/– | NA |
| P-46 | *DMD* | Exons 52-54 deletion | - | Dystrophic | +++ | + | ++ | – | – | +/– | NA |
| P-47 | *DMD* | Exons 42-54 deletion | - | Dystrophic | +++ | ++ | +++ | – | – | ++ | NA |
| P-48 | *DMD* | Exons 45-50 deletion | - | Dystrophic | ++ | +/– | ++ | – | – | +/– | NA |
| P-49 | *DMD* | Exon 45 deletion | - | Dystrophic | ++ | + | +++ | +/– | +/– | ++ | NA |
| P-50 | *DMD* | Exons 45-49 deletion | - | Dystrophic | +++ | + | +++ | ++ | ++ | ++ | NA |
| P-51 | *DMD* | Exons 3-4 duplication | - | Dystrophic | ++ | +/– | ++ | – | + | + | NA |
| P-52 | *DMD* | Exons 45-48 deletion | - | Dystrophic | ++ | + | + | + | ++ | +++ | NA |
| P-53 | *DMD* | Exons 45-47 deletion | - | Dystrophic | ++ | + | ++ | +/– | + | + | NA |
| P-54 | *DMD* | Exons 45-48 deletion | - | Dystrophic | +/– | ++ | +++ | ++ | ++ | ++ | NA |
| P-55 | *DMD* | Exons 13-47 deletion | - | Dystrophic | +++ | +++ | +++ | ++ | ++ | – | NA |

Protein expression on sections was scored into normal +++, slight reduction ++, reduction +, severe reduction +/–, and absence –. *SGCA*, NM_000023.2; *SGCB*, NM_000232.4; *SGCG*, NM_000231.2; *PMP22*, NM_000304.2; *FKRP*, NM_024301.4; *DMD*, NM_004006.2. DGC, dystrophin-glycoprotein complex; SG, sarcoglycan; Mild, mild myopathic changes; hom, homozygous; NA, not available; c.273_292del, c.273_292delCATTGGACCAAATGGCTGTG.
