# Supplementary material for: Value of muscle magnetic resonance imaging in the differential diagnosis of muscular dystrophies related to the dystrophin-glycoprotein complex
Source: Orphanet J Rare Dis. 2019 Nov 12;14:250. doi: 10.1186/s13023-019-1242-y (PMC6865054; doi:10.1186/s13023-019-1242-y)
Supplement: Supplementary file 3 — Additional file 3: Table S3. Percentages of different extent of fatty infiltration for each individual muscle in DGC-related muscular dystrophies. [file 13023_2019_1242_MOESM3_ESM.docx]

**Table S3**. Percentages of different extent of fatty infiltration for each individual muscle in DGC-related muscular dystrophies.

| Muscle fatty infiltration | Sarcoglycanopathies | | |  | LGMD2I | | |  | Dystrophinopathies | | |
| --- | --- | --- | --- | --- | --- | --- | --- | --- | --- | --- | --- |
|  | Mild (%) | Moderate (%) | Severe (%) |  | Mild (%) | Moderate (%) | Severe (%) |  | Mild (%) | Moderate (%) | Severe (%) |
| Tensor fasciae latae | 50.00 | 27.27 | 22.73 |  | 36.36 | 45.45 | 18.18 |  | 68.18 | 22.73 | 9.09 |
| Gluteus maximus | 27.27 | 36.36 | 36.36 |  | 9.09 | 54.55 | 36.36 |  | 0.00 | 77.27 | 22.73 |
| Gluteus medius | 50.00 | 22.73 | 27.27 |  | 27.27 | 54.55 | 18.18 |  | 45.45 | 54.55 | 0.00 |
| Gluteus minimus | 63.64 | 13.64 | 22.73 |  | 45.45 | 27.27 | 27.27 |  | 86.36 | 13.64 | 0.00 |
| Pectineus | 45.45 | 22.73 | 31.82 |  | 18.18 | 45.45 | 36.36 |  | 90.91 | 9.09 | 0.00 |
| Obturator externus | 50.00 | 22.73 | 27.27 |  | 27.27 | 36.36 | 36.36 |  | 95.45 | 4.55 | 0.00 |
| Obturator internus | 59.09 | 9.09 | 31.82 |  | 18.18 | 36.36 | 45.45 |  | 86.36 | 13.64 | 0.00 |
| Adductor longus | 45.45 | 9.09 | 45.45 |  | 36.36 | 18.18 | 45.45 |  | 90.91 | 9.09 | 0.00 |
| Adductor brevis | 27.27 | 27.27 | 45.45 |  | 18.18 | 18.18 | 63.64 |  | 36.36 | 40.91 | 22.73 |
| Adductor magnus | 22.73 | 9.09 | 68.18 |  | 9.09 | 18.18 | 72.73 |  | 0.00 | 50.00 | 50.00 |
| Vastus lateralis | 45.45 | 40.91 | 13.64 |  | 36.36 | 63.64 | 0.00 |  | 36.36 | 50.00 | 13.64 |
| Rectus femoris | 50.00 | 18.18 | 31.82 |  | 81.82 | 9.09 | 9.09 |  | 63.64 | 31.82 | 4.55 |
| Vastus intermedius | 36.36 | 9.09 | 54.55 |  | 18.18 | 0.00 | 81.82 |  | 63.64 | 27.27 | 9.09 |
| Vastus medialis | 27.27 | 18.18 | 54.55 |  | 18.18 | 36.36 | 45.45 |  | 45.45 | 40.91 | 13.64 |
| Sartorius | 59.09 | 31.82 | 9.09 |  | 81.82 | 18.18 | 0.00 |  | 100.00 | 0.00 | 0.00 |
| Gracilis | 68.18 | 22.73 | 9.09 |  | 81.82 | 18.18 | 0.00 |  | 100.00 | 0.00 | 0.00 |
| Biceps femoris, short head | 54.55 | 27.27 | 18.18 |  | 54.55 | 27.27 | 18.18 |  | 54.55 | 31.82 | 13.64 |
| Biceps femoris, long head | 40.91 | 27.27 | 31.82 |  | 18.18 | 27.27 | 54.55 |  | 40.91 | 45.45 | 13.64 |
| Semitendinosus | 54.55 | 27.27 | 18.18 |  | 27.27 | 27.27 | 45.45 |  | 86.36 | 13.64 | 0.00 |
| Semimembranosus | 50.00 | 36.36 | 13.64 |  | 27.27 | 45.45 | 27.27 |  | 59.09 | 31.82 | 9.09 |
| Popliteus | 86.67 | 13.33 | 0.00 |  | 100.00 | 0.00 | 0.00 |  | 88.89 | 11.11 | 0.00 |
| Tibialis anterior | 60.00 | 20.00 | 20.00 |  | 100.00 | 0.00 | 0.00 |  | 88.89 | 11.11 | 0.00 |
| Extensor hallucis and digitorum longus | 80.00 | 0.00 | 20.00 |  | 100.00 | 0.00 | 0.00 |  | 94.44 | 5.56 | 0.00 |
| Peronei | 40.00 | 46.67 | 13.33 |  | 66.67 | 0.00 | 33.33 |  | 77.78 | 22.22 | 0.00 |
| Tibialis posterior | 93.33 | 6.67 | 0.00 |  | 100.00 | 0.00 | 0.00 |  | 100.00 | 0.00 | 0.00 |
| Flexor hallucis longus | 86.67 | 0.00 | 13.33 |  | 66.67 | 33.33 | 0.00 |  | 100.00 | 0.00 | 0.00 |
| Flexor digitorum longus | 100.00 | 0.00 | 0.00 |  | 100.00 | 0.00 | 0.00 |  | 100.00 | 0.00 | 0.00 |
| Soleus | 60.00 | 40.00 | 0.00 |  | 83.33 | 16.67 | 0.00 |  | 66.67 | 27.78 | 5.56 |
| Gastrocnemius lateralis | 66.67 | 26.67 | 6.67 |  | 50.00 | 33.33 | 16.67 |  | 61.11 | 38.89 | 0.00 |
| Gastrocnemius medialis | 53.33 | 40.00 | 6.67 |  | 50.00 | 50.00 | 0.00 |  | 72.22 | 27.78 | 0.00 |

Mild fatty infiltration, scores 0–1; Moderate fatty infiltration, scores 2–3; Severe fatty infiltration, scores 4­–5. DGC, dystrophin-glycoprotein complex; LGMD, limb-girdle muscular dystrophy.
