# Supplementary material for: Value of muscle magnetic resonance imaging in the differential diagnosis of muscular dystrophies related to the dystrophin-glycoprotein complex
Source: Orphanet J Rare Dis. 2019 Nov 12;14:250. doi: 10.1186/s13023-019-1242-y (PMC6865054; doi:10.1186/s13023-019-1242-y)
Supplement: Supplementary file 4 — Additional file 4: Table S4. Further pairwise multiple comparisons between patients with different DGC-related muscular dystrophies. [file 13023_2019_1242_MOESM4_ESM.docx]

**Table S4**. Further pairwise multiple comparisons between patients with different DGC-related muscular dystrophies.

|  | Gluteus minimus | Pectineus | Obturator externus | Obturator internus | Adductor longus | Vastus intermedius | Semitendinosus | Tibialis anterior | Extensor hallucis and digitorum longus |
| --- | --- | --- | --- | --- | --- | --- | --- | --- | --- |
| Sarcoglycanopathies vs LGM2I | 0.41 | 0.32 | 0.57 | 0.14 | 0.99 | 0.27 | 0.14 | 0.04 | 0.04 |
| Sarcoglycanopathies vs Dystrophinopathies | 0.13 | 0.13 | 0.01 | 0.38 | 0.00 | 0.16 | 0.57 | 0.07 | 0.13 |
| LGM2I vs Dystrophinopathies | 0.01 | 0.01 | 0.00 | 0.01 | 0.01 | 0.01 | 0.02 | 0.68 | 0.53 |

The value in each cell was the *P* value tested by the Nemenyi test.
