# Supplementary figures and images for: Value of muscle magnetic resonance imaging in the differential diagnosis of muscular dystrophies related to the dystrophin-glycoprotein complex
Source: Orphanet J Rare Dis. 2019 Nov 12;14:250. doi: 10.1186/s13023-019-1242-y (PMC6865054; doi:10.1186/s13023-019-1242-y)

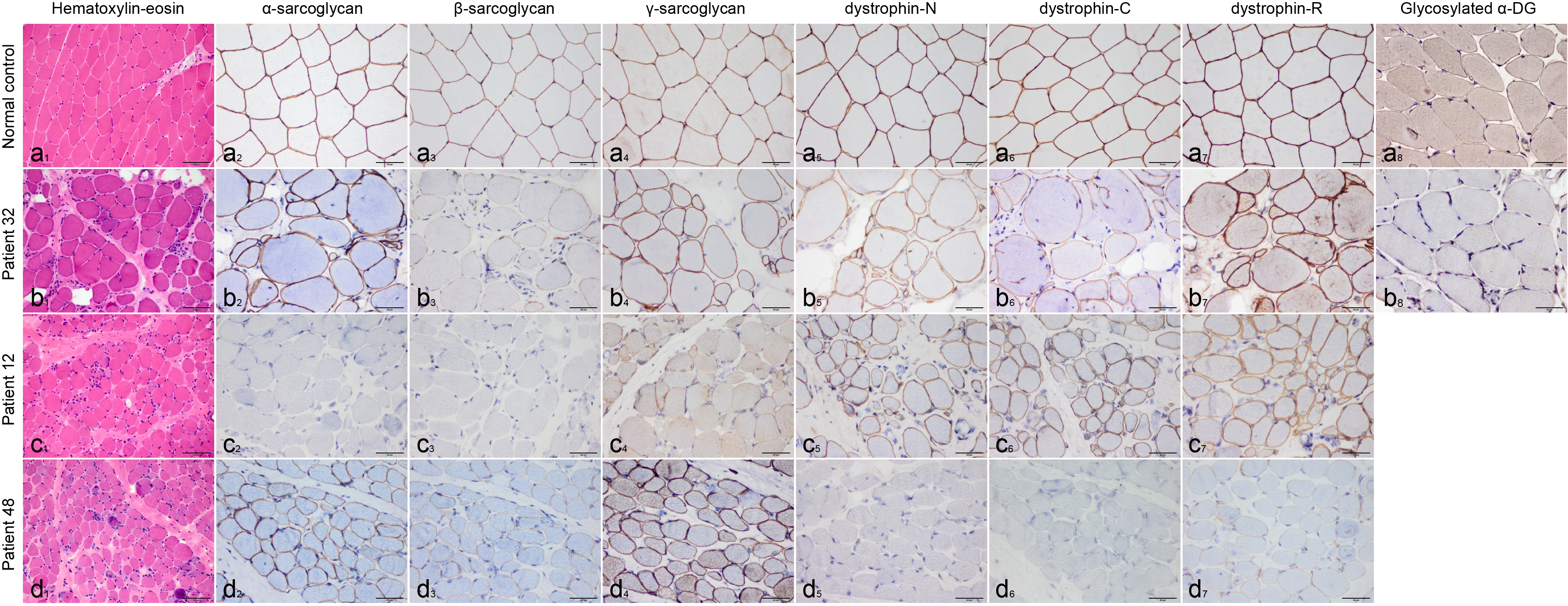

Supplement: Supplementary file 5 — Additional file 5: Figure S1. Pathologic features of patients with DGC-related muscular dystrophies. (b1, c1, and d1) Hematoxylin-eosin staining showing a dystrophic pattern in patients 32, 12, and 48. (a2–a8) A normal control showing positive staining of sarcoglycans, dystrophin, and glycosylated α-DG. (b2–b8) Patient 32 with LGMD2I showing complete deficiency of glycosylated α-DG, reduction of β-SG and dystrophin-C and -R, and positive staining of α-SG, γ-SG, and dystrophin-N. (c2–c7) Patient 12 with LGMD2D showing complete deficiency of α-SG and β-SG, reduction of γ-SG, and slight reduction of dystrophin. (d2–d7) Patient 48 with DMD showing complete deficiency of dystrophin-N and -C, severe reduction of dystrophin-R and β-SG, and slight reduction of α- and γ-SG. Hematoxylin-eosin staining (200× magnification); sarcoglycans, dystrophin, and glycosylated α-DG (400× magnification). DGC, dystrophin-glycoprotein complex; DG, dystroglycan; SG, sarcoglycan; LGMD, limb-girdle muscular dystrophy; DMD, Duchenne muscular dystrophy. [file 13023_2019_1242_MOESM5_ESM.tif]

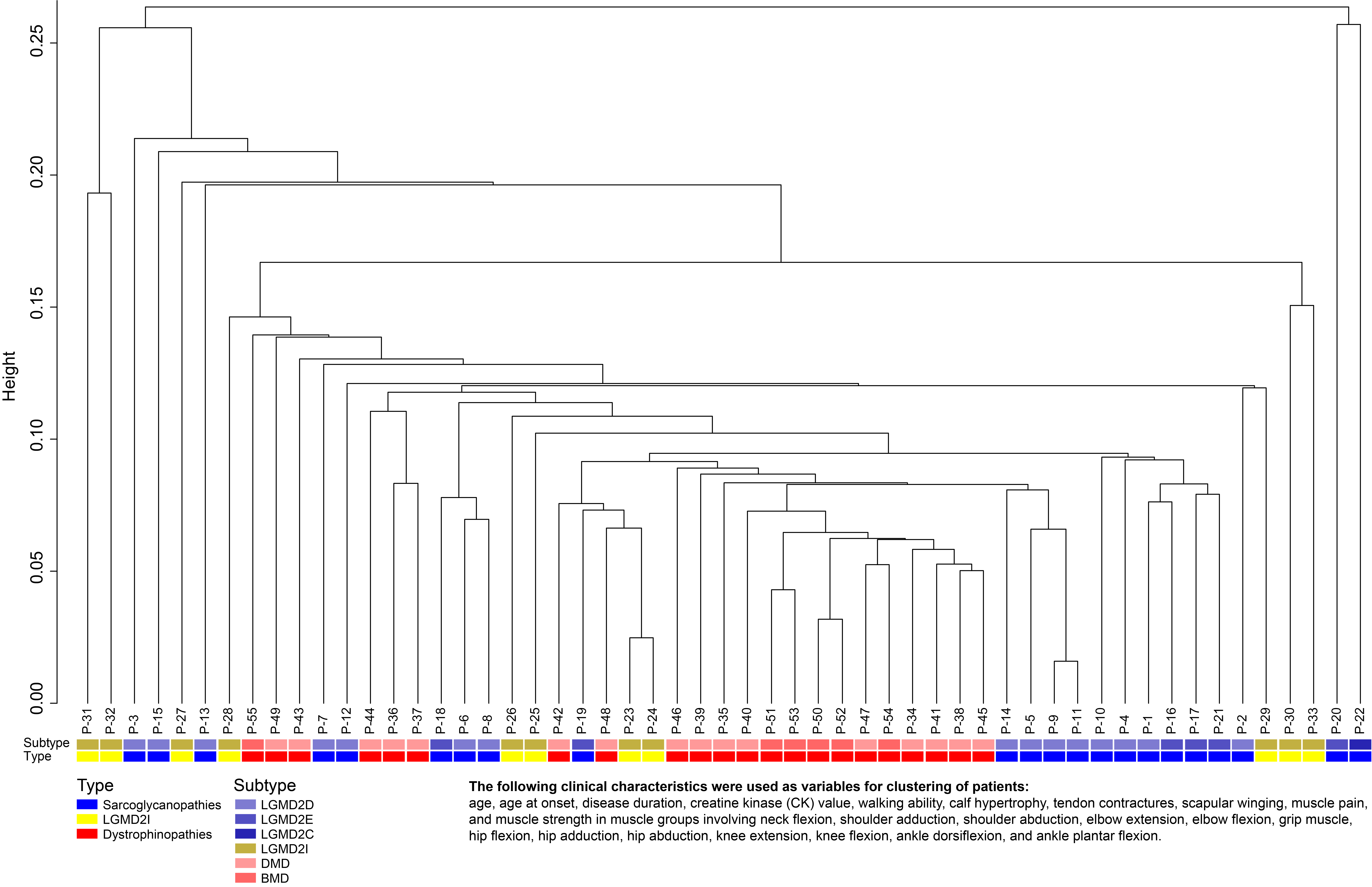

Supplement: Supplementary file 6 — Additional file 6: Figure S2. Hierarchical clustering of patients according to the clinical characteristics showing that patients did not cluster according to the genotypes. DGC, dystrophin-glycoprotein complex; LGMD, limb-girdle muscular dystrophy; DMD, Duchenne muscular dystrophy; BMD, Becker muscular dystrophy. [file 13023_2019_1242_MOESM6_ESM.tif]

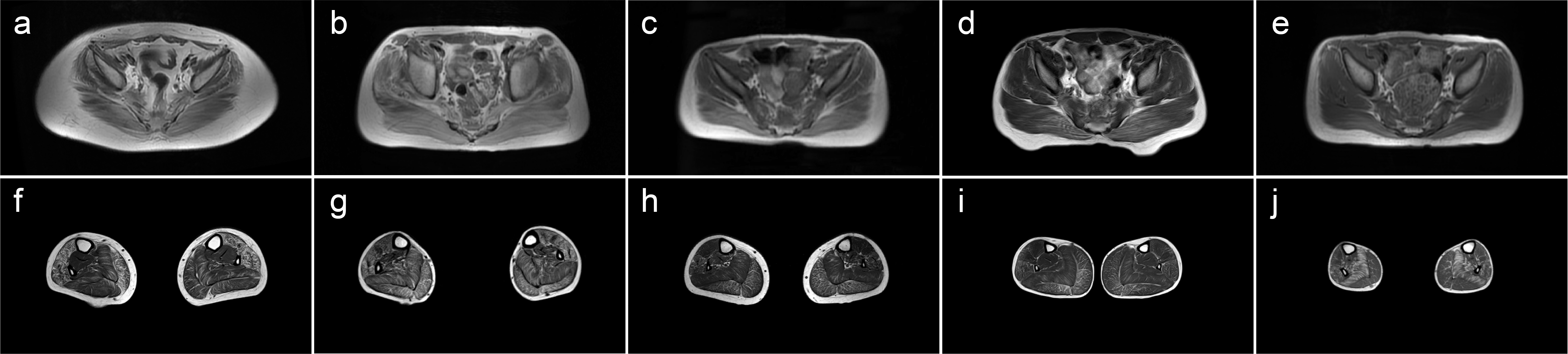

Supplement: Supplementary file 7 — Additional file 7: Figure S3. Examples of muscle fatty infiltration at the pelvis and lower leg level in DGC-related muscular dystrophies. a, patient 10, LGMD2D; b, patient 16, LGMD2E; c, patient 24, LGMD2I; d, patient 47, DMD; e, patient 54, BMD; f, patient 13, LGMD2D; g, patient 20, LGMD2E; h, patient 33, LGMD2I; i, patient 45, DMD; j, patient 55, BMD. DGC, dystrophin-glycoprotein complex; LGMD, limb-girdle muscular dystrophy; DMD, Duchenne muscular dystrophy; BMD, Becker muscular dystrophy. [file 13023_2019_1242_MOESM7_ESM.tif]
